# Safety and Efficacy of Four Different Diagnostic Catheter Curves Dedicated to One-Catheter Technique of Transradial Coronaro-Angiography—Prospective, Randomized Pilot Study. TRACT 1: Trans RAdial CoronaryAngiography Trial 1

**DOI:** 10.3390/jcm10204722

**Published:** 2021-10-14

**Authors:** Michał Chyrchel, Stanisław Bartuś, Artur Dziewierz, Jacek Legutko, Paweł Kleczyński, Rafał Januszek, Tomasz Gallina, Bernadeta Chyrchel, Andrzej Surdacki, Łukasz Rzeszutko

**Affiliations:** 1 Department of Cardiology and Cardiovascular Interventions, University Hospital, 2 Jakubowskiego Street, 30-688 Cracow, Poland; mchyrchel@gmail.com (M.C.); stanislaw.bartus@uj.edu.pl (S.B.); artur.dziewierz@uj.edu.pl (A.D.); jaanraf@interia.pl (R.J.); andrzej.surdacki@uj.edu.pl (A.S.); 2 Second Department of Cardiology, Faculty of Medicine, Institute of Cardiology, Jagiellonian University Medical College, 30-688 Cracow, Poland; chyrchelb@gmial.com; 3 Department of Interventional Cardiology, Institute of Cardiology, Jagiellonian University Medical College, John Paul II Hospital, 31-202 Krakow, Poland; jacek.legutko@uj.edu.pl (J.L.); kleczu@interia.pl (P.K.); 4 Students’ Scientific Group, Second Department of Cardiology, Jagiellonian University Medical College, 2 Jakubowskiego Street, 30-688 Krakow, Poland; tomasz.gallina@student.uj.edu.pl

**Keywords:** transradial coronaro-angiography, single-catheter technique, coronary artery disease

## Abstract

Transradial coronaro-angiography (TRA) can be performed with one catheter. We investigate the efficacy of four different DxTerity catheter curves dedicated to the single-catheter technique and compare this method to the standard two-catheter approach. For this prospective, single-blinded, randomized pilot study, we enrolled 100 patients. In groups 1, 2, 3, and 4, the DxTerity catheters Trapease, Ultra, Transformer and Tracker Curve, respectively, were used. In group 5 (control), standard Judkins catheters were used. The study endpoints were the percentage of optimal stability, proper ostial artery engagement and a good quality angiogram, the duration of each procedure stage, the amount of contrast, and the radiation dose. The highest rate of optimal stability was observed in groups 2 (90%) and 5 (95%). Suboptimal results with at least one episode of catheter fallout from the ostium were most frequent in group 1 (45%). The necessity of using another catheter was observed most frequently in group 4. The analysis of time frames directly depending on the catheter type revealed that the shortest time for catheter introduction and for searching coronary ostia was achieved in group 2 (Ultra). There were no differences in contrast volume and radiation dose between groups. DxTerity catheters are suitable tools to perform TRA coronary angiography. The Ultra Curve catheter demonstrated an advantage over other catheters in terms of its ostial stability rate and procedural time.

## 1. Introduction

Coronary angiography is still the method of choice in the diagnosis of coronary artery disease. For many years, it was performed mainly from the femoral artery. The radial approach is currently recommended as the first choice for vascular access for this purpose [1]. Transradial coronary angiography (TRA) was introduced by Campeau in 1989 [2] and Kiemeneij in 1992 [3]. In contrast to transfemoral access, TRA reduces major bleeding, access site-related vascular complications, and major adverse cardiac events and enables faster patient mobilization after the procedure [4,5,6]. TRA can be performed using two standard Judkins diagnostic catheter curves: left and right dedicated to homonymous arteries. Alternatively, TRA can be performed with one catheter designed for a single-catheter technique. Although, comparative data on the performance of different catheters are limited. Thus, in the present study, we sought to investigate the efficacy of four different catheter curves dedicated to a single-catheter technique of TRA and compare the results to the standard two-catheter approach.

## 2. Materials and Methods

**Figure 1:**
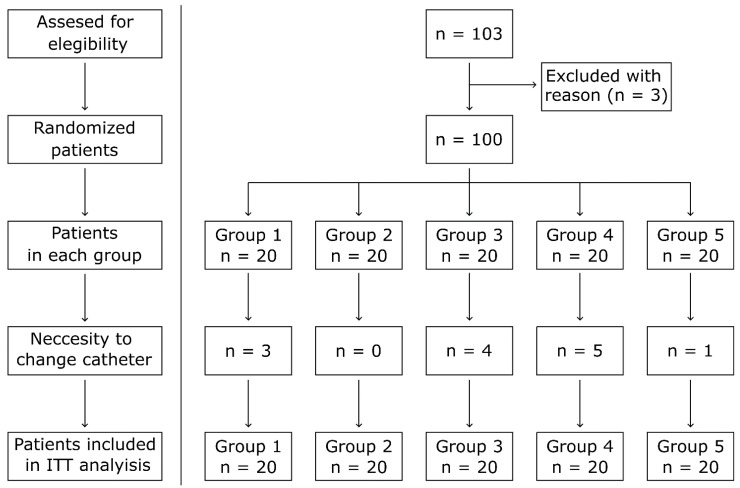
Randomization scheme. ITT—intention to treat; *n*—number of patients.

This is a prospective, single-blinded, randomized pilot study. From March 2019 to December 2020, 103 patients were screened in the Second Department of Cardiology, Jagiellonian University in Krakow. Inclusion criteria were as follows: written informed consent, stable coronary artery disease, and qualification for invasive diagnostic angiography, age >18 years, and a good pulse above the radial artery confirmed by physical examination. Exclusion criteria comprised a diagnosis of acute coronary syndrome, cardiogenic shock, previous coronary artery by-pass grafting, pregnancy, renal replacement therapy—hemodialysis with active fistula in forearm, hyperthyroidism, and previous failure of TRA. Before coronary angiography, patients were randomized using a computer-generated list into five groups. In groups 1, 2, 3, and 4, DxTerity TRA diagnosticcCatheters dedicated to the single-catheter technique of TRA angiography from Medtronic (Medtronic, Santa Rosa, CA, USA) were used. Each DxTerity catheter differs in the shape of the tip. The groups used the following catheters: group 1: Trapease Curve catheter 6F *n* = 20; group 2: Ultra Curve catheter 6F *n* = 20; group 3: Transformer Curve catheter 6F *n* = 20; group 4: Tracker Curve catheter 6F *n* = 20. Finally, in group 5 (control, standard two catheter, group), Judkins right 4.0 and Judkins left 3.5 diagnostic catheters were used, 6F *n* = 20 (Figure 1).

All procedures in the study were performed by physicians experienced in the TRA approach. TRA was successfully performed in 100 patients. Three patients were excluded from the study due to ineffective radial artery puncture and radial sheath insertion. In all excluded patients, the procedure was safely completed from the femoral artery without further complications.

Procedures were performed in a standard fashion from the right radial artery using 6F vascular sheaths. After sheath insertion, 5000 IU of unfractionated heparin was injected. Study endpoints included the percentage of catheter stability and proper engagement of coronary artery ostia during contrast injection. Ostial stability was assessed as optimal with grade 1, with proper ostial artery engagement and a good quality angiogram. Suboptimal stability was shown with grade 2, which was determined when at least one diagnostic catheter fell out from the coronary ostium and the catheter position had to be corrected. Finally, the worst stability of grade 3 was determined when ostial engagement was not achieved, and another catheter had to be introduced. The duration of each procedure stage was calculated from catheterization reports prepared by the study technician or nonoperating physician accompanying each procedure:

T1: time of beginning the procedure;

T2: time needed to introduce the diagnostic catheter, from entering the vascular sheath to reaching the ascending aorta;

T3: time needed to properly engage the ostium of the first coronary artery by the catheter positioned in the ascending aorta;

T4: time of fluoroscopy during recording the angiography of the first coronary artery;

T5: time needed to properly engage the ostium of the other coronary artery by the catheter positioned in the ascending aorta. In the standard group, the time for T5 was separated into T5a (changing Judkins catheters) and T5b (time needed to properly engage the ostium of the other coronary artery);

T6: time of fluoroscopy during recording the angiography of the second coronary artery;

T7: total procedural time.

Standard angiography projections were used: four for the left coronary artery (LCA) and two for the right coronary artery (RCA). The amount of contrast needed to find and record each coronary artery was evaluated. The total amount of contrast used during the whole procedure was measured. In all cases, contrast was injected manually. The radiation dose applied during the assessment of each coronary artery (mGy) and the total radiation dose for the whole procedure were assessed. Before angiography, the operator was obliged to declare which coronary artery would cannulated first. The frequency of change of the initial operator’s intention was assessed. The rate of necessity of using another catheter due to coronary ostium cannulation failure was also calculated. Complications related to the catheter insertion, passage through the arteries, and maneuvers in the aorta were recorded, including radial artery spasm, pain during catheter introduction, hematoma at the puncture site, upper limb hematoma, coronary artery dissection, catheter malfunction, and fracture. The serious adverse event rate was calculated, including myocardial infarction (MI), death, and repeated angiography. Basic echocardiography parameters including the left ventricular ejection fraction (EF; %); ascending aorta diameter (mm), and left ventricle maximum diameter (mm) were collected.

Statistical analysis was performed using jamovi 1.2.27 software. First, a baseline analysis, including the mean, median, standard deviation (SD) value, and assumption of normality (Shapiro–Wilk normality test), was performed. Second, to assess the statistical significance of the results, appropriate statistical tests were used. Generally, an intention-to-treat analysis was performed. Continuous variables were assessed using a one-way ANOVA (for parametric variables) or U-Mann–Whitney test, and a Kruskal–Wallis one-way ANOVA (for non-parametric variables) with post-hoc analysis was performed between each group. Nominal variables were assessed with the Chi-square test. The significance level was set at *p* < 0.05.

The study protocol was approved by the Institutional Review Board of the Jagiellonian University (approval No: 1072.6120.101.2019 issued on 24 April 2019).

## 3. Results

### 3.1. Baseline Characteristics

There was no difference among the groups with regard to age, basic anthropometric parameters, and basic echocardiographic parameters, except for the higher EF in groups 2 and 5 in compared to group 4—see Table 1.

### 3.2. Ostial Stability and Engagement in Investigated Groups

**Figure 2:**
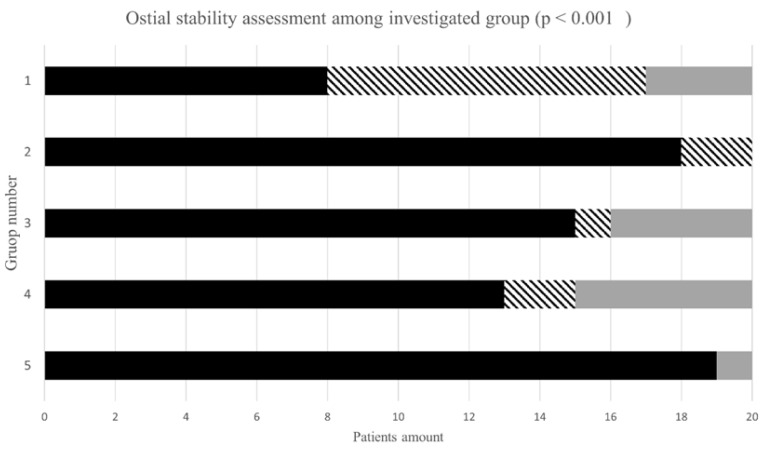
Ostial stability assessment among investigated groups. Figure legend: black—optimal stability; striped—suboptimal stability; gray—necessity to change catheter.

The highest rate of optimal stability was observed in group 2 (90%) and group 5 (95%). Suboptimal results with at least one episode of a catheter falling out from the ostium were most frequent in group 1 (45%). The necessity of usage of another catheter was observed most frequently in group 4. All results concerning catheter stability and the rate of necessity to change the catheter are presented in Figure 2 and Table S1 (Supplementary Materials).

Generally, the rate of catheter instability or necessity of catheter change was more frequently observed during the cannulation of the left coronary artery (LCA) in comparison to the right coronary artery (RCA), especially in group 1. Details are presented in Table 2.

### 3.3. Procedural Characteristic

In all groups, TRA was performed from the right radial artery. The intention to cannulate the RCA first was declared in the majority of patients irrespective of the catheter type. The exact proportions of the declared order of the cannulation of coronary arteries (right/left) among study groups were as follows: group 1: 13/7; group 2: 15/5; group 3: 14/6; group 4: 15/5; group 5: 20/0. The necessity of changing the original intention was most frequent in group 3, at four times (three times from right to left and one from left to right). In group 1 and group 4, the original intention was changed once.

### 3.4. Duration of Each Procedural Step

Time frames of each procedural stage are presented in Tables S2 and S3.

**Figure 3:**
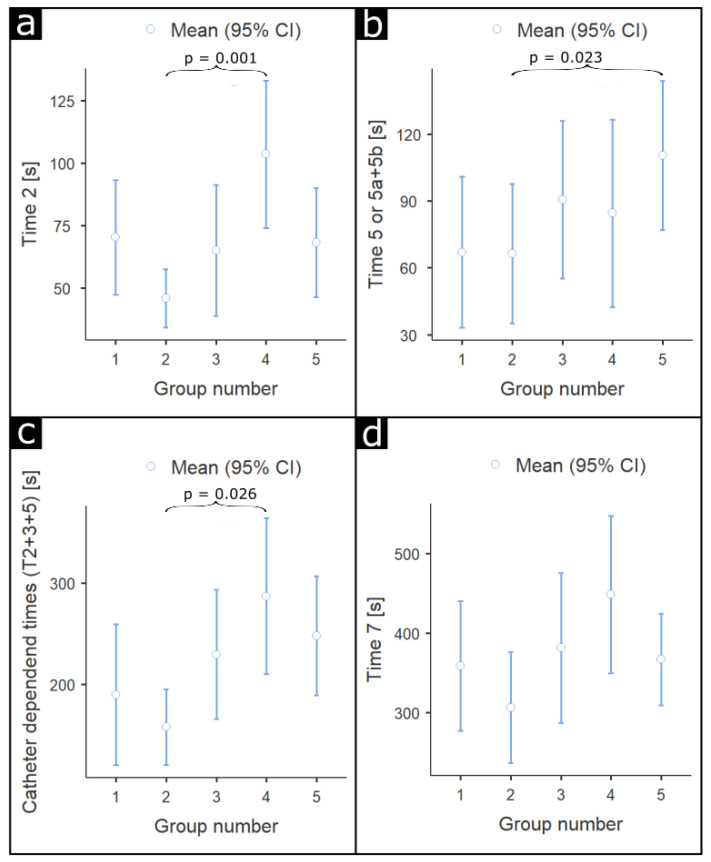
Differences in timeframes among groups. (**a**) T2 [s]: time needed to introduce the diagnostic catheter, from entering the vascular sheath to reaching the ascending aorta; (**b**) T5 [s]: time needed to properly engage the ostium of the other coronary artery by the catheter positioned in the ascending aorta. In the standard group, the time for T5 was separated into T5a (changing Judkins catheters) and T5b (time needed to properly en-gage the ostium of the other coronary artery); (**c**) T2 + T3 + T5 [s]—sum of time that directly associated with catheter type; (**d**) T7 [s]: total procedural time.

Comparing all groups, T2 was significantly shorter in group 2, with *p* = 0.005. Among particular groups, T2 was significantly shorter in group 2 then in group 4, with *p* = 0.001, and shorter in group 3 in comparison to group 4, with *p* = 0.059. Regarding T3, there were no significant differences between groups, with *p* = 0.11. There were no significant differences between groups in T4, with *p* = 0.90. T5 was shorter in group 1 (difference 43.5 s) and significantly shorter in group 2 (difference 44.1 s) in comparison to control group 5 with *p* = 0.07 and *p* = 0.023, respectively. There were no significant differences between groups in T6, with *p* = 0.11. There were also no significant differences between groups in T7, with *p* = 0.15. Analysis of timeframes directly depending on the catheter type (T2 + T3 + T5 (a + b)) revealed that the shortest time for catheter introduction and searching for coronary ostia was achieved in group 2, as shown in Figure 3.

### 3.5. Contrast Volume and Radiation Dose

Angiography of the RCA: There were no differences between study groups in terms of the contrast volume and radiation dose. Angiography of the LCA: there were no differences between study groups in contrast volume. In the post hoc analysis, the lowest radiation dose was observed in group 2, with *p* = 0.045. All results are summarized in Table 3.

### 3.6. Periprocedural Complications

Complications in all study groups were rare. There was no hematoma, coronary dissections caused by diagnostic catheters, periprocedural MI, re-PCI ± percutaneous coronary intervention), death, or catheter fracture or malfunction. Radial artery spasm was observed in one patient in group 3 and one patient in group 4—the spasm of the vessel subsided after i.a. (intra-arterial) the injection of nitroglycerin. Pain during catheter insertion was observed in one patient from groups 2, 3, and 4. In group 5, pain during catheter exchange ± from right Judkins to left one) was observed in three patients.

### 3.7. Treatment Pathway after Diagnostic Catheterization

All patients after diagnostic catheterization received optimal treatment based on the diagnostic catheterization results, patient symptoms, and preferences. Furthermore, Heart Team consultations were also taken into account, if necessary. Most of the patients received optimal pharmacological treatment—OMT ± optimal medical therapy). If invasive treatment was required, PCI ± percutaneous coronary intervention) was more often performed than CABG ± coronary artery by-pass graft). Particular information is presented in Table 4.

## 4. Discussion

**Figure 4:**
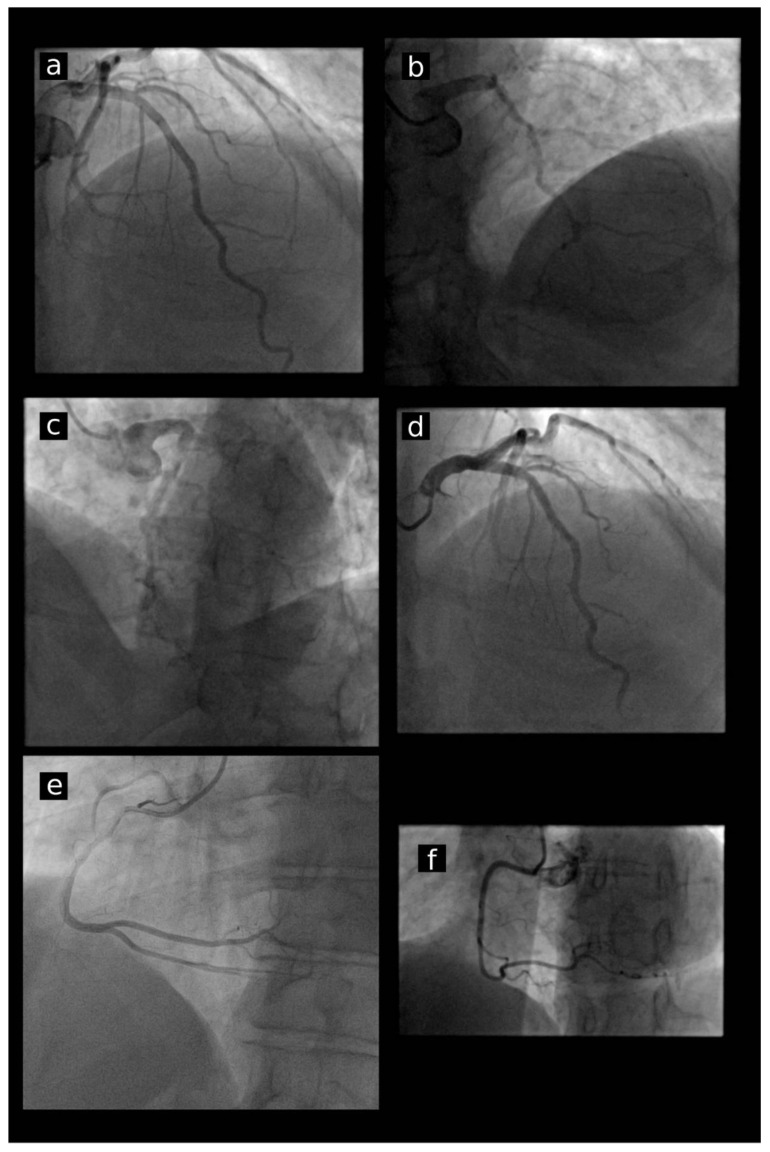
Examples of ostial stability among investigated catheters. (**a**–**c**) Instability and fall-out of investigated catheters during LCA cannulation. (**d**) Optimal stability of Ultra catheter during contrast injection to LCA, (**e**) unintentional deep intubation of RCA with Transformer catheter, (**f**) poor stability of Trapease catheter during RCA angiography. LCA—left coronary artery; RCA—right coronary artery.

Trans radial vascular access is currently the preferred access method for coronary interventions in most cathlabs [7,8]. The wide range of TRA applications in everyday practice results in a significant reduction of major bleeding and access site complications and a reduction of adverse cardiac events, and finally allows for faster patient mobilization and shorter hospitalization compared to femoral and other vascular accesses [9,10]. The main goal for introducing the single-catheter method of TRA was to avoid catheter exchange, reduce upper limb vessel mechanical irritation, and finally achieve shorter procedural times, less contrast use, and smaller radiation exposure. Chronologically, research attention was focused on the Tiger diagnostic catheter, which was designed for the one-catheter TRA concept. Originally it was assessed in a small study by Kim et al. and proved to be effective in the perfect ostial engagement of the RCA in 100% of cases and less effective in LCA ostium engagement, in 91% of cases [11]. Later, the effectiveness of the Tiger catheter was confirmed in a large study, especially in terms of engaging RCA ostium. However, almost 33% instability during left coronary angiography was demonstrated with the necessity to switch to a regular Judkins catheter to complete the procedure [12]. In the following years, the one-catheter TRA concept was investigated using TIGER II and Judkins left modified catheters [13,14,15]. In the present study, the newer generation of four different curves of DxTerity TRA diagnostic catheters from Medtronic ± Trapease, Ultra, Transformer and Tracker) dedicated to the TRA one-catheter concept were evaluated. In the study, we also observed a higher rate of successful cannulation and stability during RCA angiography in comparison with LCA for all investigated catheters ± Figure 4a–c). In previous studies, catheter instability also predominantly affected LCA. In our study, ostial stability among investigated catheters was the best for the Ultra catheter group in comparison to standard catheters ± Figure 4d). Moreover, the worst stability and highest rate of the necessity of making a catheter switch was observed in the Tracker catheter group. It is worthwhile to underline that some catheters, especially Transformer Curve, tend to deeply intubate RCA, which could increase the risk of artery dissection and require special attention from the operator ± Figure 4e). The poor stability in the present study was observed in the Trapease group ± Figure 4f). Ostial catheter stability is a very important condition for optimal performance and the adequate estimation of the angiogram. In the past, even experienced operators using the one-catheter technique complained of relatively frequent difficulties with proper catheter stable position in ostium ± non-co-axial) which was associated with frequent fall-out or poor arterial visualization during dye injection. Stability is also crucial during fractional flow reserve assessment, which is performed by some operators through diagnostic catheters.

Generally, one of the potential targets for the TRA one-catheter concept was to reduce procedural time. In the present study, we did not observe a significant difference in the total procedural time ± T7) among investigated groups. However, when times directly associated with catheter curves were analyzed ± T2, T3, T5) the shortest time periods were observed in the Ultra group. Potentially, the prolongation of the procedure through the radial artery may intensify radial spasm, reduce patient comfort, and increase the risk of complications [16,17].

According to our observations, the contrast volume was similar in all groups ± between 57–70 mL) and comparable to the study with Tiger catheters ± 65 mL), and lower than a past study with Amplatzer left catheters ± 103 mL) [18]. In other studies, the amount of contrast saved by the one-catheter strategy compared to the standard method was very small [14,15].

In cases with the one-technique catheter, pain during catheter insertion was observed very rarely. However, during TRA procedures, radial spasm can be very painful. The continuation of the procedure despite the pain could result in radial or brachial artery rupture or cross-over to the femoral artery and, in consequence, increase the risk of further complications [19]. In the present study, the rate of complications concerning radial artery reactions, hematoma rate, serious adverse events, and catheter malfunction was rare.

The major limitation of the study is the relatively small sample size. However, this is a pilot study, and investigation will be continued with a larger number of participants. Another obvious limitation is the different vascular anatomy in patients and the fact that TRA angiography was not performed in each patient using all investigated catheters. Procedures were performed by four operators, and the only vascular access site was the right radial artery. For this reason, the results cannot be automatically referenced to the procedures performed from the left radial artery.

## 5. Conclusions

DxTerity catheters dedicated to the one-catheter concept of TRA are suitable tools for performing TRA coronary angiography with a low rate of procedural complications. Different curves of diagnostic catheters seem not to be equal in terms of the effectiveness of TRA. Among the investigated catheters, the Ultra Curve catheter has demonstrated an advantage over other catheters in terms of the ostial stability rate and procedural time. Parameters which could identify the best diagnostic catheter for TRA in a single patient are still to be determined.

## Figures and Tables

**Table 1:** Baseline and echocardiographic characteristics.

Characteristics	Group 1	Group 2	Group 3	Group 4	Group 5	*p* Value
Age (years)	65.1 ± 7.8	63.1 ± 11.3	66 ± 10.1	68 ± 8.6	69.3 ± 9	0.28
Weight (kg)	84.6 ± 16.2	78.3 ± 10.1	88.5 ± 17.4	82.6 ± 20.9	77.6 ± 14.3	0.22
Height (cm)	173 ± 9.3	170 ± 9.2	171 ± 9.3	166 ± 9.2	168 ± 6.6	0.08
BMI (kg/m^2^)	28.3 ± 4.5	27.1 ± 3.1	30 ± 4.7	29.8 ± 7	27.4 ± 4.6	0.39
Men (*n* (%))	15 (75)	17 (85)	16 (80)	12 (60)	13 (65)	0.36
Diameter of aorta (mm)	35.8 ± 4.5	33.9 ± 4.0	36.1 ± 4.3	35.9 ± 6.4	35.8 ± 4.8	0.48
Left ventricle diameter (mm)	55.6 ± 9.1	50.5 ± 7.1	53.8 ± 8.2	56.7 ± 8.5	51.0 ± 7.9	0.07
EF (%)	43.6 ± 13.0	52.8 ± 12.7	46.3 ± 12.9	42.5 ± 12.0	51.8 ± 11.2	0.012 ^a^
Diabetes (*n* (%))	4 (20)	3 (15)	5 (25)	4 (20)	6 (30)	0.82
Hypertension (*n* (%))	14 (70)	13 (65)	18 (90)	15 (75)	17 (85)	0.30
PAD (*n* (%))	1 (5)	2 (10)	1 (5)	2 (10)	3 (15)	0.78
CKD (*n* (%))	2 (10)	1 (5)	3 (15)	3 (15)	5 (25)	0.45

BMI—Body Mass Index; EF—ejection fraction; PAD—peripheral artery disease; CKD—chronic kidney disease. ^a^ Kruskal–Wallis one-way ANOVA (in post-hoc analysis significant difference in group 4 vs. 2 *p* = 0.041 and group 4 vs. 5 *p* = 0.035).

**Table 2:** Rate of catheter ostial instability and rate of need for the usage of another catheter among study groups in LCA and RCA arteries.

Group	Suboptimal Ostial Stability during Cannulation of LCA and RCA (*n*)	Necessity of Catheter Change during Cannulation of LCA and RCA (*n*)
1	LCA: 8 RCA: 1	LCA: 3 RCA: 0
2	LCA: 1 RCA: 1	LCA: 0 RCA: 0
3	LCA: 1 RCA: 1	LCA: 2 RCA: 2
4	LCA: 2 RCA: 1	LCA: 2 RCA: 3
5	LCA: 0 RCA: 0	LCA: 0 RCA: 1

LCA—left coronary artery; RCA—right coronary artery.

**Table 3:** Summary of contrast volume and radiation dose during the procedure in all groups.

Amount of Contrast and Radiation	Group 1	Group 2	Group 3	Group 4	Group 5	*p* Value
RCA contrast volume (mL)	15.9 11.3	18.1 ± 9.2	23.1 ± 18.6	26.7 ± 21.3	19 ± 10.4	0.19
RCA radiation dose (mGy)	38.1 ± 25.1	29.4 ± 23.7	47 ± 27.1	41 ± 31	40.1 ± 36.6	0.23
LCA contrast volume (mL)	36.8 ± 11.3	38 ± 18.2	39.2 ± 17.5	45 ± 20.8	38.2 ± 14.4	0.71
Total radiation dose (mGy)	115 ± 71.4	67 ± 45.2	108 ± 58.5	86.9 ± 78.5	80.5 ± 40.7	0.045 ^a^
Total contrast volume (mL)	60.4 ± 32.1	57.4 ± 22.4	64.8 ± 31.4	71.8 ± 31.6	63.3 ± 30.6	0.39

LCA—left coronary artery; RCA—right coronary artery; ^a^ Kruskal–Wallis one-way ANOVA ± without significant differences between subgroups in post-hoc analysis).

**Table 4:** Treatment pathway after diagnostic catheterization.

Treatment Pathway	Group 1	Group 2	Group 3	Group 4	Group 5	*p* Value
OMT ± *n* ± %	13 ± 65	6 ± 30	10 ± 50	13 ± 65	10 ± 50	0.16
PCI ± *n* ± %	4 ± 20	11 ± 55	9 ± 45	4 ± 20	9 ± 45	0.065
CABG ± *n* ± %	3 ± 15	3 ± 15	1 ± 5	3 ± 15	1 ± 5	0.71

OMT—optimal medical therapy; PCI—percutaneous coronary intervention; CABG—coronary artery by-pass graft.

## Supplementary Materials

The following are available online at https://www.mdpi.com/article/10.3390/jcm10204722/s1, Table S1: Ostial stability assessment among investigated groups ± *p* < 0.001). Table S2: Comparison of time frames between single catheter groups and control group. And Table S3: Comparison of time frames in each group.
