# Supplementary material for: Safety and Efficacy of Four Different Diagnostic Catheter Curves Dedicated to One-Catheter Technique of Transradial Coronaro-Angiography—Prospective, Randomized Pilot Study. TRACT 1: Trans RAdial CoronaryAngiography Trial 1
Source: J Clin Med. 2021 Oct 14;10(20):4722. doi: 10.3390/jcm10204722 (PMC8541157; doi:10.3390/jcm10204722)
Supplement: Supplementary file 1 [file jcm-10-04722-s001.zip › jcm-1420233-supplementary.pdf]

Supplementary Materials

Table S1. Ostial stability assessment among investigated groups ($p < 0.001$).

Group number		Stability			
		**Optimal**	**Suboptimal**	**Necessity to change catheter**	**Total**
**1.**	Observed	8	9	3	20
	% in row	40.0 %	45.0 %	15.0 %	100.0 %
**2.**	Observed	18	2	0	20
	% in row	90.0 %	10.0 %	0.0 %	100.0 %
**3.**	Observed	15	1	4	20
	% in row	75.0 %	5.0 %	20.0 %	100.0 %
**4.**	Observed	13	2	5	20
	% in row	65.0 %	10.0 %	25.0 %	100.0 %
**5.**	Observed	19	0	1	20
	% in row	95.0 %	0.0 %	5.0 %	100.0 %
**Total**	Observed	73	14	13	100
	% in row	73.0 %	14.0 %	13.0 %	100.0 %

Table S2. Comparison of time frames between single catheter groups and control group.

	**Single catheter groups. (groups 1-4) N=80**	**Control group N=20**	**p value**
**T2 [s]**	71 ± 54	68 ± 47	0.89
**T3 [s]**	68 ± 69	69 ± 75	0.93
**T4 [s]**	50 ± 35	41 ± 18	0.52
**T5 / T5a+T5b [s]**	77 ± 76	111 ± 71	0.008[a]
**T6 [s]**	108 ± 90	79 ± 45	0.087
**T7 [s]**	374 ± 189	367 ± 123	0.705
**T2+T3+T5 [s]**	216 ± 142	248 ± 125	0.18

[a] U-Mann-Whitney test.

Table S3. Comparison of time frames in each group.

	**Group 1**	**Group 2**	**Group 3**	**Group 4**	**Group 5**	**p value**
**T2 [s]**	70 ± 49	46 ± 35	65 ± 56	104 ± 63	68 ± 47	0.005[a]
**T3 [s]**	52 ± 72	46 ± 32	748 ± 56	99 ± 94	69 ± 75	0.11
**T4 [s]**	48 ± 24	53 ± 40	56 ± 54	43 ± 19	41 ± 18	0.90
**T5/T5a+T5b [s]**	67 ± 73	66 ± 67	91 ± 76	85 ± 90	111 ± 71	0.055[b]
**T6 [s]**	121 ± 63	95 ± 71	96 ± 56	119 ± 58	79 ± 45	0.11
**T7 [s]**	358 ± 174	306± ± 149	381 ± 201	448 ± 211	367 ± 123	0.15
**T2+T3+T5 [s]**	190 ± 148	158 ± 80	230 ± 136	287 ± 164	248 ± 125	0.017[c]

[a] Kruskal-Wallis One-Way ANOVA (significant difference in group 2 vs. 4; p = 0.001); [b] Kruskal-Wallis One-Way ANOVA (significant difference in group 2 vs. 5; p = 0.023); [c] Kruskal-Wallis One-Way ANOVA (significant difference in group 2 vs 4; p = 0.026).
